# A comparison of antibiotic resistance reports in pharmacovigilance databases and conventional surveillance across “One Health”

**DOI:** 10.3389/fpubh.2026.1758180

**Published:** 2026-05-15

**Authors:** Joseph Mitchell, Manju Purohit, Pinelopi Lundquist, Camilla Westerberg, Cecilia Stålsby Lundborg

**Affiliations:** 1 Department of Global Public Health, Health Systems and Policy: Improving Use of Medicines, Karolinska Institutet, Stockholm, Sweden; 2 Uppsala Monitoring Centre, Uppsala, Sweden; 3 Department of Pathology, R.D. Gardi Medical College, Ujjain, India

**Keywords:** antibiotic resistance, EudraVigilance Veterinary, GLASS, One Health, pharmacovigilance, VigiBase

## Abstract

**Introduction:**

Pharmacovigilance has been highlighted as a potential additional source of information to supplement conventional antibiotic resistance surveillance. However, it is not known how well the potential cases of antibiotic resistance identified in pharmacovigilance databases replicate those captured in traditional surveillance, either in human medicine or across “One Health.”

**Methods:**

Cases of antibiotic resistance captured by traditional surveillance were taken from publicly available data for both humans and animals. The data sources used were the WHO Global Antibiotic Resistance and Use Surveillance System (GLASS), and the joint European Food Safety Authority (EFSA) and European Centre for Disease Prevention and Control (ECDC) report, respectively, for humans and animals. Pharmacovigilance data were taken from VigiBase, the WHO global database of adverse event reports, for humans, and from EudraVigilance Veterinary for animals. Potential antibiotic resistance cases were identified using previously reviewed search criteria. No suitable data sources were identified for environmental health. Data were grouped by Anatomical Therapeutic Chemical (ATC) class to the third level and by continent (only Europe was used for animals). Likelihood ratio tests of logistic regression models with and without an interaction between the ATC class and database were then used to compare the reported antibiotic distribution.

**Results:**

For VigiBase, there were 26,086 reports from 91 countries identified. There was a consistent statistically significant difference between the distribution of cases for at least one ATC third-level grouping. The search of EudraVigilance Veterinary identified 1,010 cases from 18 countries. Again, there was a consistent, statistically significant difference between the distribution of the cases for at least one ATC third-level grouping compared with EFSA/ECDC data.

**Discussion:**

There was more complete surveillance for humans compared to animals, with the study restricted to Europe only for animals but conducted globally for humans based on the availability of data. The absence of an appropriate data source for environmental health further highlights the need to improve surveillance in environmental health. The statistically significant differences between the antibiotic distribution should be investigated further, as this could improve the understanding of the relative strengths and weaknesses of pharmacovigilance databases as a potential supplementary tool.

## 1 Introduction

Antibiotic resistance is a prominent global health problem that has warranted high levels of interest, including at the 2024 UN General Assembly. There is a recognition that antibiotic resistance requires action from a “One Health” perspective that acknowledges the interlinked nature of humans, animals, and environmental health (1, 2). This global threat has also prompted coordinated action from the World Health Organization (WHO), the World Organisation for Animal Health (WOAH), the Food and Agriculture Organization (FAO), and the United Nations Environment Programme (UNEP) (3). Furthermore, integrated antibiotic resistance surveillance, which incorporates humans, animals, and environmental health, is highlighted as a top research priority (3, 4).

Extensive efforts have been undertaken to develop surveillance systems, particularly for humans. The WHO established the Global Antibiotic Resistance and Use Surveillance System (GLASS) in 2015 to collate data on antibiotic resistance, antibiotic use, and invasive fungal infections (5). In 2022, 127 countries, territories, or areas participated in GLASS (5). There is no equivalent global antibiotic resistance surveillance system for animals, with the majority of the surveillance systems focusing on national surveillance (6). The European Food Safety Authority (EFSA) and European Centre for Disease Prevention and Control (ECDC) produce a joint annual report that includes antibiotic resistance measurements taken from healthy food-producing animals, primarily within the European Union (7). A complementary surveillance system that focuses on pathogenic infections in animals is being developed for potential use (8, 9). There is no environment-based global antibiotic resistance surveillance system, and at regional and national levels, these systems are typically less well developed (10), although the need for potential methods and sources has been explored (2, 11) and progress has been made.

The resistance of *Escherichia coli* to third-generation cephalosporins is used by the WHO as one of two indicators of progress toward the UN Sustainable Development Goals (12), and resistance levels in *E. coli* is used as the indicator in the EFSA and ECDC joint reports on antimicrobial resistance (13). *E. coli* is intrinsically sensitive to almost all antibacterial agents, is a significant commensal bacterium, and has a great ability to act as an antibiotic resistance reservoir with the capability to receive and spread resistance genes (14).

Despite the differences in the antibiotic resistance surveillance capabilities across “One Health,” there remains a need for integrated antibiotic resistance surveillance systems (4). However, even within human-based antibiotic resistance surveillance, there remain areas for improvement, with a bias towards hospital data and other methodological considerations that can inhibit interpretation (2, 11). To reach populations across the world, it has been suggested that antibiotic resistance surveillance should incorporate alternative, complementary data sources (2), with pharmacovigilance discussed as one of these potential sources (15).

Pharmacovigilance, “the science of activities relating to the detection, assessment, understanding, and prevention of adverse effects or any other medicine/vaccine-related problem,” (16) has the ability to capture reports across the scope of “One Health” (50). In contrast to antibiotic resistance surveillance, which tracks the confirmed number of tests, pharmacovigilance is based primarily on collecting reports of suspected adverse events. However, the maturity of pharmacovigilance systems varies across these areas; however, the most comprehensive systems are found in human health, followed by animal health, while those related to environmental pharmacovigilance are the least developed.

The potential role of pharmacovigilance as a tool in antibiotic resistance surveillance is still in the early stages of exploration. However, the speculated roles include the identification of cases (17–20), product quality issues (21), and improper antibiotic use (15, 18). In the field of animal health, identifying potential cases may be indicated by a lack of effectiveness (20, 22). While human health has received the most attention, there has been a description of reporting across the areas of “One Health” (51). This study aims to compare the information within traditional antibiotic resistance surveillance systems with that within pharmacovigilance databases across “One Health.”

## 2 Methods

### 2.1 Data sources

#### 2.1.1 Human data sources

To analyze global antibiotic resistance data, the WHO GLASS data dashboard (accessible from https://worldhealthorg.shinyapps.io/glass-dashboard/) was used. This dataset uses the most up-to-date estimate of global antibiotic resistance, with data from 2022. The results are presented as the number of interpretable antibiotic susceptibility tests from bacteriologically confirmed infections and the number of tests that were resistant. Further information on the methodology may be found in the GLASS 2022 report (5). Data were used for bloodstream, gastrointestinal, gonorrhoeal, and urinary tract infections from all geographical regions for the following bacteria: *Acinetobacter* spp. (bloodstream), *E. coli* (bloodstream and urinary tract), *Klebsiella pneumoniae* (bloodstream and urinary tract), *Salmonella* spp. (bloodstream and gastrointestinal), *Shigella* spp. (gastrointestinal), *Neisseria gonorrhoeae* (gonorrhoeal), and *Streptococcus pneumoniae* (bloodstream). *Staphylococcus aureus* was not included in the results as it was only collected as an indicator for the UN Sustainable Development Goals. The antibiotics included in the GLASS surveillance were selected due to them being either common first line treatment options, surrogate markers for other frequently used medicines, or due to resistance to the antibiotic of a specific pathogen being a particular concern (23). Data from all antibiotics that were tested for susceptibility against the included infectious agents were used in this study.

For human pharmacovigilance data, this study analyzed VigiBase, the WHO global database of adverse event reports for medicines and vaccines. VigiBase receives reports of suspected adverse events from more than 180 members of the WHO Programme for International Drug Monitoring, and the database contains around 40 million reports (24). VigiBase receives reports from multiple sources, which may differ depending on the country of origin. These reports can be received from, but it is not limited to, healthcare professionals, consumers, and the pharmaceutical industry, and the probability that the suspected adverse effect is related to the drug is not the same in all cases. Data were retrieved from a dataset from 1 April, 2024, and all reports from the first in 1967 until the end of 2022 were used. MedDRA®, the Medical Dictionary for Regulatory Activities terminology, is the dictionary used to code suspected adverse events, and within the dictionary hierarchy, the Preferred Term (PT) is most commonly used for analysis. The search comprised all reports that had an antibiotic, as a suspected or interacting medicine, in combination with a pre-determined set of PTs that potentially represented antibiotic resistance. Antibiotics were identified using the Anatomical Therapeutic Chemical (ATC) classification, maintained by the WHO Collaborating Centre for Drug Statistics Methodology (25). ATC J01 (antibacterial agents for systemic use) was used to define antibiotics. The PTs used in the search to find potential cases of antibiotic resistance have been described in-depth in previous research and found to be adept at identifying reports of interest (see Supplementary Table 1) (26).

By March 2025, four countries, areas, or territories contributed to the GLASS, either toward the antibiotic use or antibiotic resistance data that were not full or associate members of the WHO PIDM. A total of 41 countries, areas, or territories are full or associate members of the WHO PIDM that do not contribute to the GLASS.

#### 2.1.2 Animal data sources

Due to the lack of global data, antibiotic resistance data for animals were taken from the EFSA and ECDC joint report on antimicrobial resistance in humans, animals, and food, with data from 2021 to 2022 (7). This report on zoonotic (*Campylobacter* spp. and *Salmonella* spp.) and indicator (*E. coli*) bacteria is based on data submitted by the European Union Member States and four non-Member States, who voluntarily provided to the relevant authorities. After the United Kingdom left the European Union by the end of 2020, it was considered as a non-Member state, but the EFSA did receive information from Northern Ireland in accordance with the withdrawal agreement, so the United Kingdom (with data only from Northern Ireland) was included as a Member State (7). The antibiotic resistance tests were performed using the samples from five food-producing animals (pigs, cattle under 1 year, broiler chickens, laying hens, and turkeys) (7). This study used data collected for *E. coli* and *C.* spp. from food and commensal samples from the 27 Member States and the United Kingdom. Since data on the number of resistant tests performed for *S.* spp. were unavailable, these data were excluded. The antibiotics used in the report for each pathogen are shown in Supplementary Table 2.

EudraVigilance Veterinary was used as the animal-based pharmacovigilance source. The database is maintained by the European Medicines Agency and is aligned with VigiBase, and the reports represent the reporter’s opinion and are the suspected adverse events (27). The database collects reports on medicinal products that are licensed for use in veterinary care within the European Economic Area (EEA), although reports are not limited to originating only from countries within the EEA. To best match the antibiotic resistance surveillance undertaken in the EFSA/ECDC report, this study used reports only from the European Union and United Kingdom. EudraVigilance Veterinary uses the Veterinary Dictionary for Drug Regulatory Authorities (VedDRA) to code the reported adverse events. The VedDRA follows a hierarchy similar to MedDRA® and similarly uses PTs as the typical level of analysis. The PT “Lack of Effect” was used to identify possible cases of antibiotic resistance, as was utilised in previous research (50) and is likely how cases of ABR would present in animal healthcare (22). The Anatomical Therapeutic Chemical Veterinary (ATCvet) classification was used to identify antibiotics; this was done by using ATCvet QJ01 in EudraVigilance, which is the animal equivalent of ATC J01. According to the ATC classification, the ATCvet classification systems is maintained by the WHO Collaborating Centre for Drug Statistics Methodology. The two classifications are developed in close association with one another to enable comparison in drug utilization studies (28). All reports up to the end of 2022 were used in this study.

#### 2.1.3 Environmental data sources

No established database was identified for reporting environmental antibiotic resistance or for pharmacovigilance purposes. A previous review found scant reports related to environment-related PTs in combination with antibiotics in both VigiBase and EudraVigilance Veterinary (50). Therefore, an analysis of environment-based data was not performed.

### 2.2 Analysis

The data from the GLASS dashboard included for each country, the total number of tests, the total number of resistant tests, and the percentage of resistant tests for each antibiotic. The results were grouped by ATC at the third level (such as ATCJ01A – tetracyclines), by continent as defined by the UN Geoscheme (29) (Africa, Americas, Asia, Europe, and Oceania), by the infection type (all infections and bloodstream infections), and by income-level of the country according to the 2022 World Bank classification (30).

For the animal-based data, the total number of tests, the number of resistant outcomes, and the percentage of resistant tests were calculated for each ATCvet third level within QJ01, the ATCvet group equivalent to ATC J01. These data were calculated for all organisms. No continental differentiation was performed as all reports originated from Europe.

For the pharmacovigilance data, the reporting country and the individual antibiotics data, along with their respective ATC or ATCvet codes, were extracted. As more than one antibiotic, and thus ATC code, can be included in each report, analysis was conducted on the number of individual antibiotics reported rather than the number of reports within the pharmacovigilance databases, to ensure all antibiotics were captured. For VigiBase data, the reporting country was grouped into continents, as per the UN geoscheme (29), and by the income-level of the country according to the 2022 World Bank classification (30). The reports were also grouped according to a previous study (“Probable” and “Possible”), which separated the PTs used in the search based on the likelihood of representing antibiotic resistance (26). However, there was a statistically significant difference between these groups in, among other factors, the likelihood of being a case of antibiotic resistance, the income-level of the country, and the antibiotic classification according to the AWaRe categories (26).

For the EudraVigilance Veterinary data, the reporting country was differentiated into EU Member States plus the United Kingdom and non-EU Member States. For both databases, the data were analyzed using all reports for each ATC or ATCvet third-level group and then by their geographical distribution as defined earlier. The EudraVigilance Veterinary data were also analyzed based on animal species deemed to a food-producing animal (such as cattle, chicken, duck, fish, goat, Guinea fowl, partridge, pheasant, pig, quail, sheep, and turkey), when the study species matched the species used in the EFSA/ECDC report (such as cattle, chicken, pig, and turkey).

The total number of tests, the total positive/negative tests, and the percentage of positive tests were calculated for each ATC third-level group from traditional ABR data sources. For pharmacovigilance data, the number of reported antibiotics was also recorded to the ATC third level. The percentage of reporting was calculated using the total number of antibiotics reported in the potential ABR cases in VigiBase as the denominator. Using the total number of potential antibiotic resistance reports as the denominator, rather than all reports with an antibiotic, was selected to remove the impact of unequal reporting of antibiotics for other adverse events. When comparing the data sources, the ATC third-level groups that were not represented in both datasets were excluded for both the percentage calculations and the logistic regression models.

Logistic regression models were used to compare the levels of antibiotic resistance reported between the different data sources. These models only compared within the same “One Health” field, preferably VigiBase versus GLASS and EudraVigilance Veterinary versus EFSA/ECDC. The third level of the ATC or ATCvet classification was used in the logistic regression models. The outcomes of the logistic regression models were the number of positive tests and negative tests (calculated as the number of individual antibiotic substances reported that were not in that ATC third level of the pharmacovigilance data). The predictors included the interaction between the ATC or ATCvet group and the database and another separate model without an interaction. For humans, separate logistic regression models were run on all data, based on continent, income-level of the country (high-income countries and low- and middle-income countries), likelihood of the VigiBase search terms to represent antibiotic resistance (“Probable” and “Possible”), and the reports in VigiBase only from the last 5 years of data collection up to 2022. For animals, separate models were run on all data, based on only food-producing animals, matching animal species, and all reports from EudraVigilance Veterinary from the last 5 years of data collection up to 2022. Likelihood ratio tests were conducted between models for each model group (interaction included versus interaction not included) to test for statistically significant difference between at least one ATC third-level group between databases.

## 3 Results

### 3.1 Results based on humans

The results of the antibiotic susceptibility testing were available for 20 unique antibiotic substances, from 87 countries from all five UN continents. In VigiBase, the search identified 26,086 reports with 38,476 individual antibiotics within these reports. There were 254 unique antibiotic substances, with submission of reports from 91 countries. The distribution based on the income level of the countries is shown in Table 1. There was a dominance of reporting from high-income countries for both the number of tests performed and the number of antibiotics reported in VigiBase. The total number of susceptibility tests reported to the GLASS was 21,368,340.

**Table 1:** Distribution of tests performed in GLASS and individual antibiotics reported in VigiBase, using income-level of the reporting country.

World Bank income level	Number of susceptibility tests in GLASS				Number of individual antibiotics reported in VigiBase	
	All infectious sources		Bloodstream infections			
	*n*	%	*n*	%	*n*	%
High-income	16,605,129	77.7	2,691,452	75.5	32,898	85.5
Middle-income	4,723,933	22.1	863,116	24.2	5,546	14.4
Low-income	39,278	0.2	8,313	0.2	32	0.1

GLASS, Global antibiotic resistance and use surveillance system.

**Figure 1:**
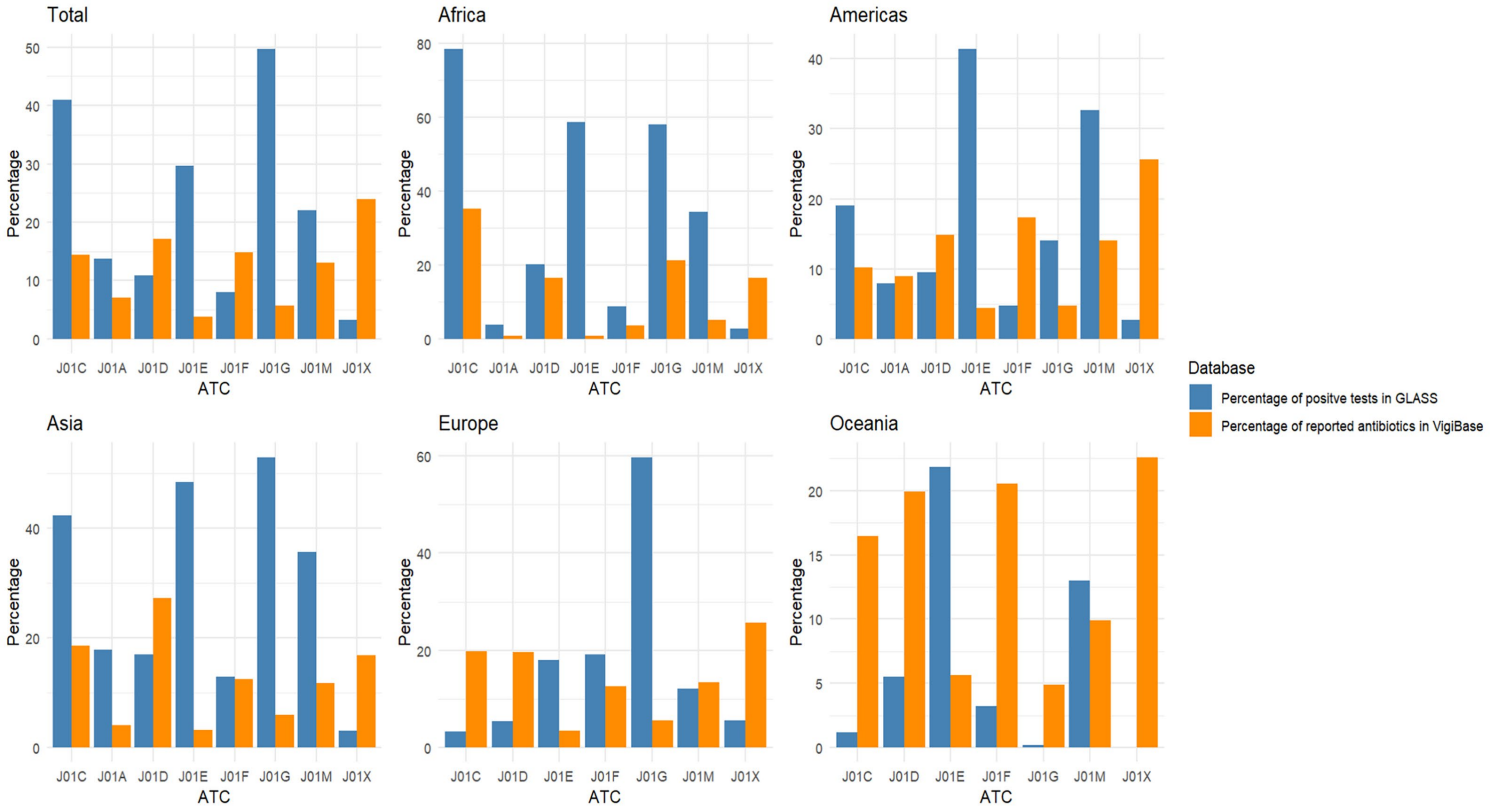
Percentage of resistant tests in GLASS for all sources of infection and the percentage of reported antibiotics in potential cases of antibiotic resistance in VigiBase for each ATC third group level, for all reports together and then grouped by continent. Number of antibiotics included from VigiBase reports for each graph – Total, *n* = 38,160; Africa, *n* = 1,599; America, *n* = 22,174; Asia, *n* = 3,964; Europe, *n* = 9,238; and Oceania, *n* = 637. Number of tests reported to GLASS for each graph – Total, *n* = 21,368,340; Africa, *n* = 640,617; America, *n* = 2,508,409; Asia, *n* = 7,945,582; Europe, *n* = 9,645,449; and Oceania, *n* = 628,283. ATC, Anatomical herapeutic hemical; J01A, Tetracyclines; J01C, Beta-lactam antibacterials, penicillin; J01D, Other beta-lactam antibacterials; J01E, Sulfonamides and trimethoprim; J01F, Macrolides, lincosamides, and streptogramins; J01G, Aminoglycoside antibacterials; J01M, Quinolone antibacterials; J01X, Other antibacterials.

The percentage of positive tests from the GLASS and the percentage of reported individual antibiotics in VigiBase for each ATC third-level group for all reported microorganisms are shown in Figure 1. The figure shows that within the GLASS data, there were some large discrepancies between the percentages of positive tests reported based on continent. For example, the level of resistance reported to ATC J01C in Africa was 78.5%, compared to 3.3% in Europe. The relative percentage of antibiotics reported in VigiBase for potential antibiotic resistance cases, however, was generally more consistent, with the percentage of antibiotics reported within J01C being 35.3 and 19.9% for Africa and Oceania, respectively. However, the data were calculated as a percentage of all reports, so it is not surprising that this resulted in a more equal distribution due to the overall percentage being limited to 100%. A full breakdown of test results in the GLASS data is shown in Supplementary Table 2.

There were some consistencies of identified antibiotic resistance across the continental regions. For example, the ATC groups with the highest percentage of positive tests in GLASS, included J01C (beta-lactam antibacterials, penicillin), J01E (sulfonamides and trimethoprim), J01G (aminoglycoside antibacterials), and J01M (quinolone antibacterials) for all reports, and reports from Africa, America, and Asia. However, the results are not true for Europe and the Oceania, they still had J01G (aminoglycoside antibacterials) and J01E (sulfonamides and trimethoprim) as the groups with the highest percentage of resistance, respectively. Despite their being a relatively more equal distribution of reported antibiotics by ATC third-level groups in the VigiBase, there were some observable patterns. The relative percentage of reporting of antibiotics in VigiBase, the ATC groups J01C (beta-lactam antibacterials, penicillin), J01D (other beta-lactam antibacterials), J01F (macrolides, lincosamides, and streptogramins), and J01X (other antibacterials) were among the groups with the highest percentage of reported antibiotics across the continents.

**Figure 2:**
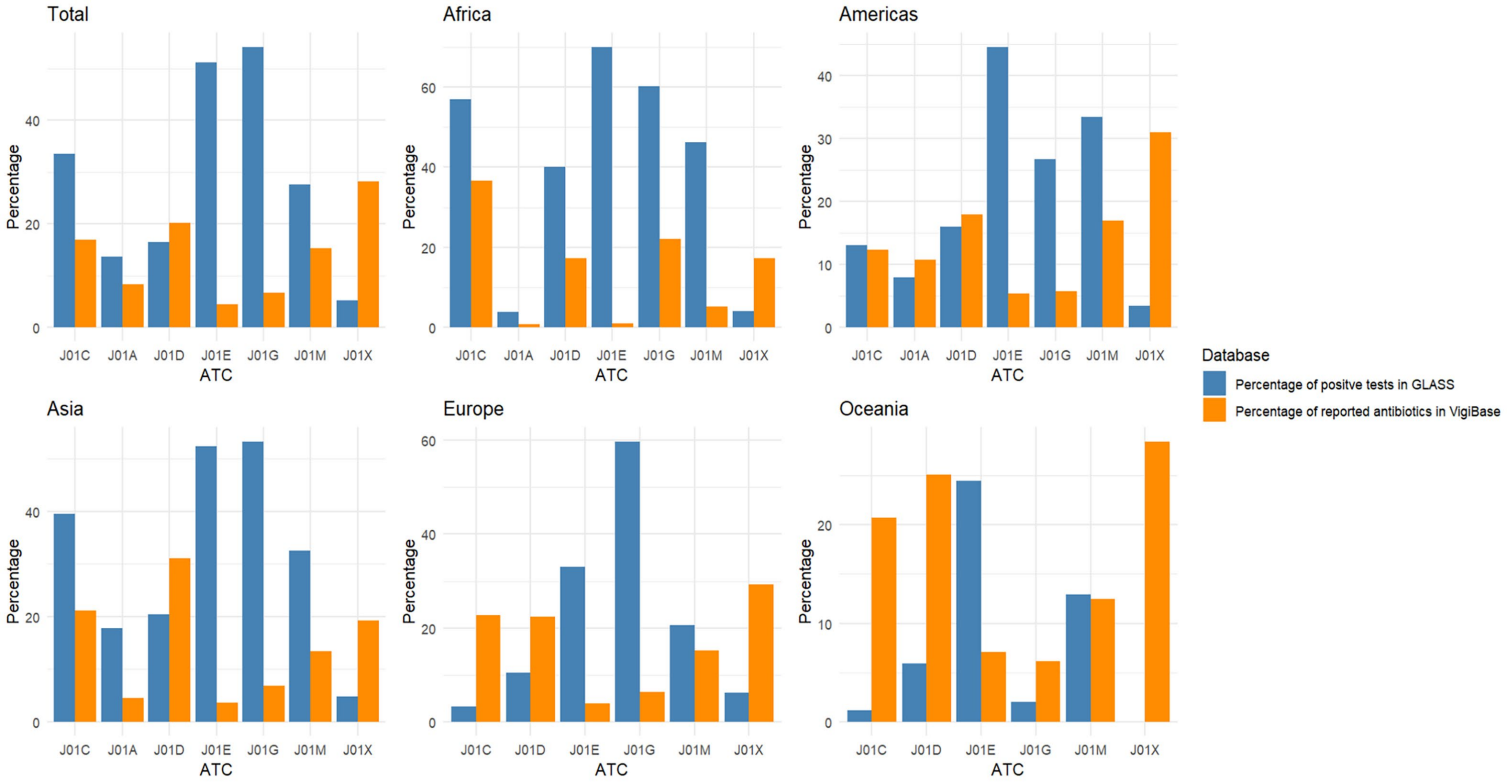
Percentage of resistant tests in GLASS for bloodstream infections and the percentage of reported antibiotics in potential cases of antibiotic resistance in VigiBase for each ATC third-group level, for all reports together and then grouped by continent. Number of antibiotics included from VigiBase reports for each graph – Total, *n* = 3*2,480; Africa, n* = 1,542; America, *n* = 18,331; Asia, *n* = 3,471; Europe, *n* = 8,082; and Oceania, *n* = 506. Number of tests reported to GLASS for each graph – Total, *n* = 3,562,881; Africa, *n* = 158,823; America, *n* = 254,708; Asia, *n* = 1,644,290; Europe, *n* = 1,455,477; and Oceania, *n* = 49,583. ATC, Anatomical Therapeutic Chemical; J01A, Tetracyclines; J01C, Beta-lactam antibacterials, penicillin; J01D, Other beta-lactam antibacterials; J01E, Sulfonamides and trimethoprim; J01G, Aminoglycoside antibacterials; J01M, Quinolone antibacterials; J01X, Other antibacterials.

Figure 2 shows the distribution of positive tests in GLASS for bloodstream infections and the percentage of reported individual antibiotics in VigiBase. Similar patterns of positive test results in GLASS were seen between bloodstream infections and all infection sources, with a wide variation between continents and ATC groups. In an earlier example, the percentage of positive tests for ATC J01C (beta-lactam antibacterials, penicillin) in Africa decreased by 57.0%, but this remained high compared to the 3.3% positive tests in Europe. As there were different ATC third-level groups included due to the antibiotics reported in the GLASS data, the percentages of reported antibiotics within ATC J01 in Africa was 36.6% when compared with 22.7% in Europe. There were in general, similarities between bloodstream infections and all infections for the ATC groups with the highest percentage of resistant tests reported.

There was at least one ATC group with a statistically significant difference in distribution for all reports grouped together, and for each continent separately (all *p*-values = <2.2^−16^). This remains true when reports limiting to only bloodstream infections reported to GLASS, when separating by income level, VigiBase search terms, and last 5 years of reports in VigiBase (see Supplementary Figures 1–3).

### 3.2 Results based on animals

The search of EudraVigilance Veterinary identified a total of 1,010 reports, with 1,329 individual antibiotics. There were 81 unique antibiotic substances, and reports were received from 18 countries. By using only the food-producing animals, the number of reports wasd 783, with 1,036 individual antibiotics reported. The total of unique antibiotics reported was 66 with reports from 17 countries. For reports matching only with species, there were 950 reports, with the same amount of antibiotics reported. The total of unique antibiotics was 65 with the reports from 16 countries. The total number of susceptibility test results in the EFSA/ECDC joint report was 152,247.

**Figure 3:**
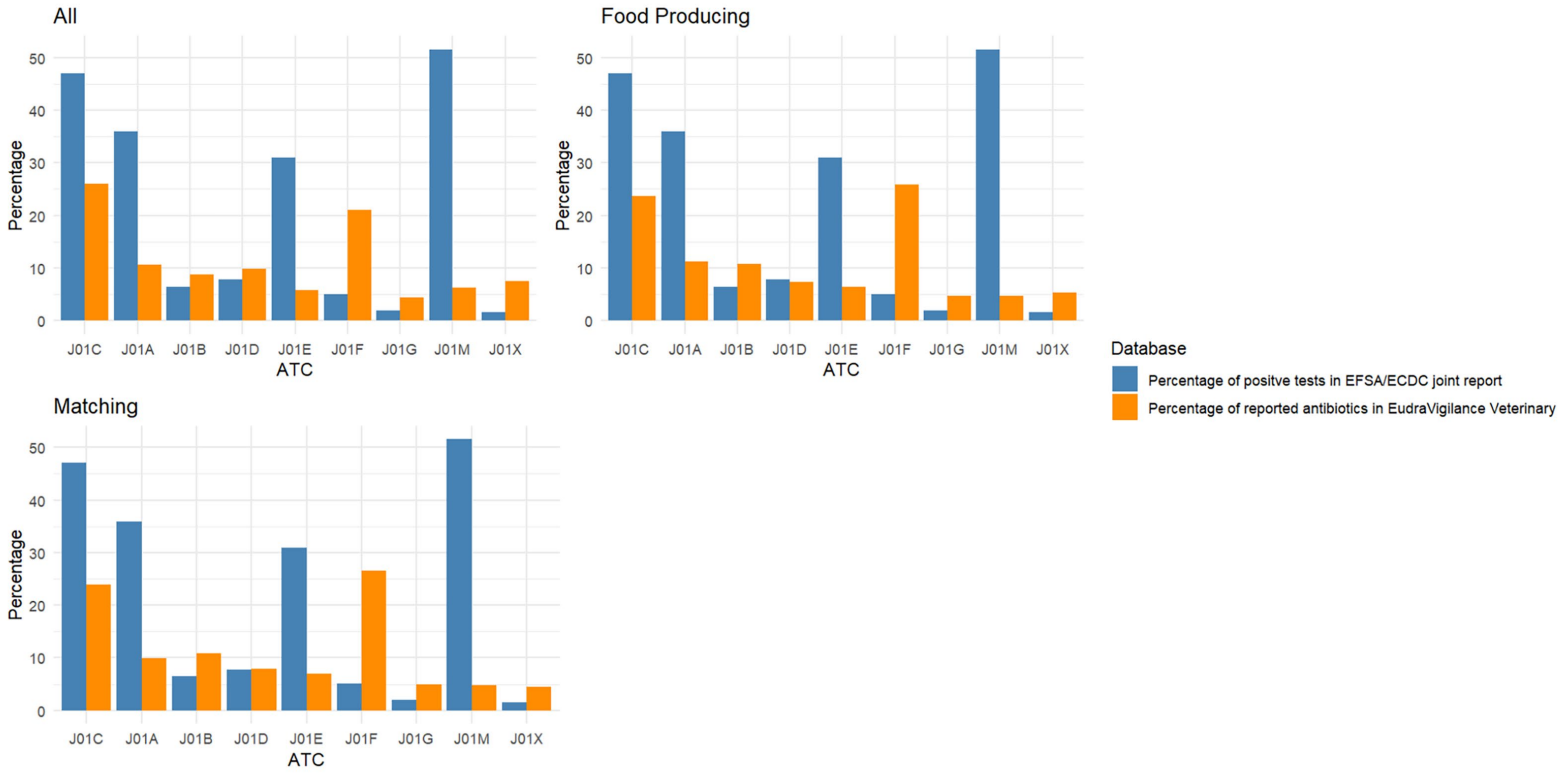
Percentage of resistant tests in the EFSA/ECDC joint report and the percentage of reported antibiotics in potential cases of antibiotic resistance in EudraVigilance veterinary, for each ATC third group level, for all reports and for food-producing and matched animals. Number of antibiotics included from EudraVigilance reports for each graph – All *n* = 1,281; Food producing species *n* = 992; Matching species *n* = 906. Number of tests reported to EFSA/ECDC, *n* = 152,247. ATC, Anatomical Therapeutic Chemical; J01A, Tetracyclines; J01B, Amphenicols; J01C, Beta-lactam antibacterials, penicillin; J01D, Other beta-lactam antibacterials; J01E, Sulfonamides and trimethoprim; J01F, Macrolides, lincosamides, and streptogramins; J01G, Aminoglycoside antibacterials; J01M, Quinolone antibacterials; J01X, Other antibacterials.

Figure 3 shows the percentage of positive tests in the EFSA/ECDC joint report and the relative percentage of individual reported antibiotics for potential cases of antibiotic resistance in EudraVigilance Veterinary. There were similarities in the percentage of positive tests in all the different constellations of animal groups, with the highest percentage of positive tests in EFSA/ECDC data, including ATCvet groups J01A (tetracyclines), J01C (beta-lactam antibacterials, penicillin), J01E (sulfonamides and trimethoprim), and J01M (quinolone antibacterials). In the EudraVigilance data, the highest percentage of reported antibiotics were in the ATCvet groups, including J01A (tetracyclines), J01C (beta-lactam antibacterials, penicillin), and J01F (macrolides, lincosamides, and streptogramins).

There was a statistically significant difference (all *p*-values = <2.2^−16^) for at least one of the ATC groups for all animals and when limiting the EudraVigilance Veterinary data to include only food-producing species, matching animal species, and reports from the last 5 years (see Supplementary Figure 4).

## 4 Discussion

To the best of the authors’ knowledge, this is the first study that compared the reporting of potential antibiotic resistance cases in pharmacovigilance databases and those identified through traditional antibiotic resistance surveillance. This study highlights that, for both humans and animal health, there are significant differences between the potential antibiotic resistance cases reported to pharmacovigilance databases and those captured using the conventional antibiotic resistance surveillance.

For humans, this difference has remained true when looking at the reporting across continents and there was also reasonable consistency of ATC groups that had the highest percentage of resistant tests or number of antibiotics reported for their respective databases. The statistically significant difference was also consistent in the continents that had relatively differing patterns of reporting, such as Europe and Oceania for VigiBase. This difference may provide vital information in the potential utility of pharmacovigilance databases as a supplementary tool in antibiotic resistance surveillance. However, this difference may be influenced by different factors.

First, the infections captured by traditional antibiotic surveillance may be skewed towards severe infections from urban settings due to their reliance on data from tertiary surveillance (2, 31). This bias has, in part, led to the call for the use of alternative data sources (2, 32), with pharmacovigilance discussed as a supplementary option as the system may capture reports from rural and low-income settings (33). There is also potential data skewing in VigiBase, the previous study showed that a more specific search for antibiotic resistance cases had a higher number of antibiotics from the Reserve antibiotic classification (26). This could indicate more severe infections (34), although this is not the intended use of the AWaRe classification (35) and there may be other external factors that influence reporting (36). However, this study has included a less specific search for potential antibiotic resistance cases in VigiBase, which contributes the majority of the reports, which are less severe infections and more likely to be reported by non-healthcare professionals (26). There is also an inherent difference between what is being captured by the databases, with antibiotic resistance databases collecting confirmed cases of antibiotic resistance to specific infections, from specific centers. However, pharmacovigilance data may include both confirmed cases of antibiotic resistance and cases of a clinical suspicion of antibiotic resistance, or other causes of antibiotic treatment failure and not all cases are equal in their likelihood of causality or in this case being a case of antibiotic resistance (36). When the VigiBase data were filtered to include only the more specific search only, and the less specific search only (“Probable” and “Possible”), the statistically significant difference remained.

Second, while both datasets include reports from around the world, this study cannot determine whether the intra-continental distribution of received test results for antibiotic resistance surveillance and reports in pharmacovigilance databases is equal. This can be influenced by the relative resources available, the maturity of the antibiotic resistance surveillance and pharmacovigilance systems and the reporting patterns of individual countries and regions. This study also analyzed the human databases, by filtering the dataset so that it included only high-income countries and low- and middle-income countries. For both tests, the statistically significant difference remained. Generally, there are differences between antibiotic use and resistance in high-, low-, and middle-income countries. There can also be significant differences between countries on the same geographical continent when it comes to antibiotic use and resistance. The consistency of the statistically significant difference between the databases, which also included when restricting the VigiBase reports to the previous 5 years, strengthens the likelihood that there is a true difference between the databases when it comes to the distribution of at least one ATC group at the third level.

For animals, this study conducted analyses ensuring that reports from the EudraVigilance Veterinary were from the same countries as used in the EFSA/ECDC report. This study was also able to do separate analyses for food-producing animals and even matching animal species. For all these different analyses there was a statistically significant difference for at least one ATC group when comparing between the two databases. This is an important consideration when considering the results as the scopes of the databases are different. The EFSA/ECDC report focuses on animals being used for food production and EudraVigilance Veterinary derives reports primarily from veterinary care, and there are differences in antibiotic usage in the food-producing animals and the pet animals (37, 38). This difference is still an important consideration that could affect the results, but by doing these different analyses this study has mitigated as much as possible for these differences and increases the likelihood that the observed difference between the databases is due to a difference in the reports collected rather than purely the difference in scope. Similarly to the human data, the statistically significant difference remained when limiting the EudraVigilance Veterinary reports to the past 5 years.

There was a reasonable consistency in the most reported antibiotic groups, when this study carried out different subsets of the pharmacovigilance data. Antibiotics within J01C (beta-lactam antibacterials, penicillin) and J01D (other beta-lactam antibacterials) are amongst the most used antibiotics in human healthcare (39). Similarly, for animal health in Europe, the most commonly used antibiotics belong the groups ATC J01C (beta-lactam antibacterials, penicillin) and J01A (tetracyclines) (40) and these were among the most reported antibiotics in the EudraVigilance Veterinary. However, for both human and animal health, there was not always a clear link between antibiotic usage and reporting in pharmacovigilance databases. For example, J01X (other antibacterials) and J01F (macrolides, lincosamides, and streptogramins) were among the most reported for humans and animals, respectively, but they are not among the antibiotic groups that are most prescribed (39, 40). As discussed earlier, these differences could be in part due to the differences in aims and methods of data collection. These differences in aims and data collection mean that perhaps the identified differences at this level is not unexpected, despite there being some similarities between the two datasets for some of the antibiotic groups. Identifying the reason for the statistically significant differences, for at least one ATC third-level group, and where the similarities lie could lead to a better understanding of whether incorporating pharmacovigilance data alongside traditional antibiotic resistance surveillance is beneficial. Previous studies have found that individual case reports may contain valuable information that is likely to be missed by traditional antibiotic resistance surveillance, potentially triggering local investigations (17, 26). Further exploration of the relationship between the groups of antibiotics reported to pharmacovigilance databases, the use of antibiotics, and antibiotic resistance patterns may provide further information with regards to the differences between traditional ABR databases and pharmacovigilance. Such further research may be performed in a local or regional level, given that there is considerable variation in the use of antibiotics between countries (40, 41), and also variation in the pharmacovigilance and antibiotic resistance surveillance systems. Local investigating would make it easier to assess how the individual cases can provide important supplementary information and whether existing pharmacovigilance systems adds value to the antibiotic resistance surveillance systems (26).

The absence of databases, and therefore analysis, related to environmental exposure highlights the dearth of information for both antibiotic resistance and pharmacovigilance. This has also been shown in other studies of environment-related terms reported to international pharmacovigilance databases (51). This is despite the increasing importance of recognizing the impact of environmental exposure in both antibiotic resistance and pharmacovigilance (42, 43). However, there have been recent publications highlighting the increasing ability to measure and predict temporospatial antibiotic resistance genes in various environments (44–48). These developments could be used as an initial foothold to improve the monitoring of pharmaceuticals in the environment in the broader sense.

The compared data for traditional antibiotic surveillance and pharmacovigilance databases were collected and collated in different ways. Therefore, this study was not able to directly compare similar data, but for pharmacovigilance data, a more proxy measure was used, i.e., the relative reporting of antibiotics in the potential cases rather than the number of positive and negative tests for antibiotic susceptibility. The differences in data collection also meant that the study was not able to investigate whether there was a general correlation between reported antibiotics in pharmacovigilance databases and the percentage of resistance cases tested by traditional antibiotic surveillance. This would have provided valuable information as to highlight similarities and differences beyond one ATC third-level group, and ensuring data collection allows such analyses is an important step in future research. But we were able to highlight if there is a difference for at least one ATC group for each analysis. This comparison is extremely relevant as it highlights the difference in data collected by the different methods and further examination of these differences will allow greater interpretation of any potential utility of pharmacovigilance data in antibiotic resistance surveillance. Such future steps include more specifically identifying where the differences lie, and which of the potential underlying cause or causes of the differences contribute to creating these differences from the perspective of pharmacovigilance. As stated earlier, given the variation in surveillance systems, both for antibiotic resistance and pharmacovigilance, this is perhaps best implemented on a local level. Furthermore, the identification of antibiotic resistance cases in VigiBase has been assessed to be reasonably accurate (26), although this has not been investigated for EudraVigilance Veterinary. The location of the infection, the infective organism, and the antibiotics used, and how they are used, can impact the success of treatment and any possible development of antibiotic resistance. There is important missing information with regards to how antibiotics are used, with no information on antibiotic usage in the antibiotic resistance surveillance data. These datasets do benefit from an inherent inclusion of the indication for antibiotic use, or antimicrobial susceptibility testing. It is possible to capture the indication, route of administration, and doses of each antibiotic within pharmacovigilance case reports, but they are typically not captured fully as in the case reports (49).

## 5 Conclusion

This study has shown that there are significant differences between the percentage of antibiotic resistance in GLASS for antibiotic groups and the number of reports for each antibiotic group in potential cases of antibiotic resistance in pharmacovigilance databases. These differences were consistent across the analyses performed and for both humans and animals. Further understanding of the differences may allow clearer interpretation of how pharmacovigilance can best be incorporated as a supplement to traditional antibiotic resistance. The absence of information for environment-related resistance highlights the necessity for improving surveillance within both antibiotic resistance surveillance and pharmacovigilance.

## Data Availability

The datasets presented in this article are not readily available because the data that support the findings of this study are not publicly available. Access to the VigiBase data is restricted based on the conditions for access within the WHO Programme for International Drug Monitoring. Subject to these conditions, some of the data are available from the authors on reasonable request. Data from EudraVigilance Veterinary were requested and received from the European Medicines Agency. Requests to access the datasets should be directed to joseph.mitchell@ki.se.

## References

[1] One Health High-Level Expert Panel (OHHLEP) Adisasmito WB Almuhairi S Behravesh CB Bilivogui P Bukachi SA . One health: a new definition for a sustainable and healthy future. PLoS Pathog. (2022) 18:e1010537. doi: 10.1371/journal.ppat.1010537, 35737670 PMC9223325

[2] Okeke IN de Kraker MEA Van Boeckel TP Kumar CK Schmitt H Gales AC . The scope of the antimicrobial resistance challenge. Lancet. (2024) 403:2426–38. doi: 10.1016/S0140-6736(24)00876-6, 38797176

[3] FAO, UNEP, WHO, WOAH. A one Health Priority Research Agenda for Antimicrobial Resistance. Geneva: World Health Organization (2023).

[4] Aenishaenslin C Häsler B Ravel A Parmley J Stärk K Buckeridge D. Evidence needed for antimicrobial resistance surveillance systems. Bull World Health Organ. (2019) 97:283–9. doi: 10.2471/BLT.18.218917, 30940985 PMC6438253

[5] World Health Organization. Global Antimicrobial Resistance and use Surveillance system (GLASS) Report: 2022. Geneva: World Health Organization (2022).

[6] Simjee S McDermott P Trott DJ Chuanchuen R. Present and future surveillance of antimicrobial resistance in animals: principles and practices. Microbiol Spectrum. (2018) 6. doi: 10.1128/microbiolspec.ARBA-0028-2017, 30003869 PMC11633600

[7] Authority (EFSA) EFS, European Centre for Disease Prevention and Control (ECDC). The European Union summary report on antimicrobial resistance in zoonotic and indicator bacteria from humans, animals and food in 2021–2022. EFSA J. (2024) 22:e8583. doi: 10.2903/j.efsa.2024.8583 38419967 PMC10900121

[8] Mader R Damborg P Amat JP Bengtsson B Bourély C Broens EM . Building the European antimicrobial resistance surveillance network in veterinary medicine (EARS-vet). Euro Surveill. (2021) 26:2001359. doi: 10.2807/1560-7917.ES.2021.26.4.2001359, 33509339 PMC7848785

[9] Lagrange J Amat JP Ballesteros C Damborg P Grönthal T Haenni M . Pilot testing the EARS-vet surveillance network for antibiotic resistance in bacterial pathogens from animals in the EU/EEA. Front Microbiol. (2023) 14:1188423. doi: 10.3389/fmicb.2023.1188423 37283921 PMC10239921

[10] Collineau L Bourély C Rousset L Berger-Carbonne A Ploy MC Pulcini C . Towards one health surveillance of antibiotic resistance: characterisation and mapping of existing programmes in humans, animals, food and the environment in France, 2021. Euro Surveill. (2023) 28:2200804. doi: 10.2807/1560-7917.ES.2023.28.22.2200804, 37261729 PMC10236929

[11] Larsson DGJ Flach CF Laxminarayan R. Sewage surveillance of antibiotic resistance holds both opportunities and challenges. Nat Rev Microbiol. (2023) 21:213–4. doi: 10.1038/s41579-022-00835-5, 36470999 PMC9734844

[12] World Health Organization. Sustainable development goals (SDGs) AMR indicator. (2025). Available online at: https://www.who.int/data/gho/data/themes/topics/global-antimicrobial-resistance-surveillance-system-glass/sustainable-development-goals-amr-indicator [Accessed July 15, 2025]

[13] Mader R Bourély C Amat JP Broens EM Busani L Callens B . Defining the scope of the European antimicrobial resistance surveillance network in veterinary medicine (EARS-vet): a bottom-up and one health approach. J Antimicrob Chemother. (2022) 77:816–26. doi: 10.1093/jac/dkab462, 35022739 PMC8864999

[14] Poirel L Madec JY Lupo A Schink AK Kieffer N Nordmann P . Antimicrobial Resistance in *Escherichia coli*. Microbiol Spectrum. (2018) 6:10.1128/microbiolspec.arba-0026–2017. doi: 10.1128/microbiolspec.ARBA-0026-2017, 30003866 PMC11633601

[15] Habarugira JMV Figueras A. Pharmacovigilance network as an additional tool for the surveillance of antimicrobial resistance. Pharmacoepidemiol Drug Saf. (2021) 30:1123–31. doi: 10.1002/pds.5249, 33864401

[16] WHO. Pharmacovigilance. (2020). Available online at: https://www.who.int/teams/regulation-prequalification/regulation-and-safety/pharmacovigilance [Accessed August 2, 2020]

[17] Habarugira JMV Härmark L Figueras A. Pharmacovigilance data as a trigger to identify antimicrobial resistance and inappropriate use of antibiotics: a study using reports from the Netherlands pharmacovigilance Centre. Antibiotics (Basel). (2021) 10:1512. doi: 10.3390/antibiotics10121512, 34943724 PMC8698598

[18] Mhaidat NM Al-Azzam S Banat HA Jaber JM Araydah M Alshogran OY . Reporting antimicrobial-related adverse drug events in Jordan: an analysis from the VigiBase database. Antibiotics (Basel). (2023) 12:624. doi: 10.3390/antibiotics12030624, 36978491 PMC10044927

[19] Cagnotta C Zinzi A Gargano F Liguori V Campitiello MR Perrella A . Can pharmacovigilance data represent a potential tool for early detection of the antibiotic resistance phenomenon? Pharmacoepidemiol Drug Saf. (2024) 3:350–64. doi: 10.3390/pharma3040024

[20] De Briyne N Gopal R Diesel G Iatridou D O’Rourke D. Veterinary pharmacovigilance in Europe: a survey of veterinary practitioners. Vet Rec Open. (2017) 4:e000224. doi: 10.1136/vetreco-2017-000224, 28848652 PMC5554794

[21] Jasovsky D Aagaard H Zorzet A. Antimicrobial resistance - an overlooked adverse event. Uppsala Reports; (2017). Available online at: https://who-umc.org/media/2775/web_uppsalareports_issue74.pdf (Accessed April 15, 2024).

[22] Bengtsson B Greko C. Antibiotic resistance—consequences for animal health, welfare, and food production. Ups J Med Sci. (2014) 119:96–102. doi: 10.3109/03009734.2014.901445, 24678738 PMC4034566

[23] Hope M Kiggundu R Tabajjwa D Tumwine C Lwigale F Mwanja H . Progress on implementing the WHO-GLASS recommendations on priority pathogen-antibiotic sensitivity testing in Africa: a scoping review. Wellcome Open Res. (2024) 9:692. doi: 10.12688/wellcomeopenres.23133.1, 39931110 PMC11809157

[24] Uppsala Monitoring Centre. A global collaboration for patient safety. (2022). Available online at: https://who-umc.org/about-the-who-programme-for-international-drug-monitoring/about-the-who-pidm/ [Accessed August 2, 2022]

[25] WHO Collaborating Centre for Drug Statistics Methodology. ATCDDD - structure and principles. (2024). Available online at: https://atcddd.fhi.no/atc/structure_and_principles/#Therapeu [Accessed November 12, 2024]

[26] Mitchell J Westerberg C Purohit M Lundquist P Lundborg CS. The enhancing role of pharmacovigilance to conventional antibiotic resistance surveillance: cross-sectional identification and analysis of reports of antibiotic resistance in VigiBase. Int J Infect Dis. (2025) 158:107947. doi: 10.1016/j.ijid.2025.107947, 40480533

[27] EudraVigilance European database of suspected adverse drug reaction reports. (2024). Available online at: https://www.adrreports.eu/vet/en/index.html [Accessed May 7, 2024]

[28] ATCDDD. Guidelines for ATCvet classification. (2025). Available online at: https://atcddd.fhi.no/atcvet/atcvet_index_and_guidelines/guidelines_for_atcvet_classifica/ [Accessed September 13, 2025]

[29] UN Statistics Division. UNSD — methodology. (2025). Available online at: https://unstats.un.org/unsd/methodology/m49/ [Accessed January 31, 2025]

[30] Hamadeh N Van Rompaey C Metreau E Grace Eapen S. New World Bank Country Classifications by Income Level: 2022–2023. Washington: World Bank Blogs (2022).

[31] Iskandar K Molinier L Hallit S Sartelli M Hardcastle TC Haque M . Surveillance of antimicrobial resistance in low- and middle-income countries: a scattered picture. Antimicrob Resist Infect Control. (2021) 10:63. doi: 10.1186/s13756-021-00931-w 33789754 PMC8011122

[32] Ashley EA Shetty N Patel J van Doorn R Limmathurotsakul D Feasey NA . Harnessing alternative sources of antimicrobial resistance data to support surveillance in low-resource settings. J Antimicrob Chemother. (2019) 74:541–6. doi: 10.1093/jac/dky487, 30544186 PMC6406030

[33] Habarugira JMV Figueras A. Antimicrobial stewardship: can we add pharmacovigilance networks to the toolbox? Eur J Clin Pharmacol. (2021) 77:787–90. doi: 10.1007/s00228-020-03035-3, 33196869

[34] Abdelsalam Elshenawy R Umaru N Aslanpour Z. WHO AWaRe classification for antibiotic stewardship: tackling antimicrobial resistance – a descriptive study from an English NHS foundation trust prior to and during the COVID-19 pandemic. Front Microbiol. (2023) 14:1298858. doi: 10.3389/fmicb.2023.1298858 38146447 PMC10749484

[35] Saleem Z Sheikh S Godman B Haseeb A Afzal S Qamar MU . Increasing the use of the WHO AWaRe system in antibiotic surveillance and stewardship programmes in low- and middle-income countries. JAC Antimicrob Resist. (2025) 7:dlaf031. doi: 10.1093/jacamr/dlaf031, 40110554 PMC11919820

[36] Upppsala Monitoring Centre. UMC VigiBase Caveat document. (2025). Available online at: https://who-umc.org/media/yzpnzmdv/umc_caveat.pdf [Accessed October 11, 2025]

[37] Prescott JF. Antimicrobial use in food and companion animals. Anim Health Res Rev. (2008) 9:127–33. doi: 10.1017/s1466252308001473, 18983721

[38] Bandyopadhyay S Samanta I. Antimicrobial resistance in Agri-food chain and companion animals as a re-emerging menace in post-COVID epoch: low-and middle-income countries perspective and mitigation strategies. Front Vet Sci. (2020) 7:620. doi: 10.3389/fvets.2020.00620, 33195500 PMC7581709

[39] Klein EY Impalli I Poleon S Denoel P Cipriano M Van Boeckel TP . Global trends in antibiotic consumption during 2016–2023 and future projections through 2030. Proc Natl Acad Sci USA. (2024) 121:e2411919121. doi: 10.1073/pnas.2411919121, 39556760 PMC11626136

[40] European Medicines Agency. European Sales and Use of Antimicrobials for Veterinary Medicine: Annual Surveillance Report for 2023. Luxembourg: Publications Office (2025).

[41] Hsia Y Sharland M Jackson C Wong ICK Magrini N Bielicki JA. Consumption of oral antibiotic formulations for young children according to the WHO access, watch, reserve (AWaRe) antibiotic groups: an analysis of sales data from 70 middle-income and high-income countries. Lancet Infect Dis. (2019) 19:67–75. doi: 10.1016/s1473-3099(18)30547-4, 30522834

[42] Larsson DGJ Flach CF. Antibiotic resistance in the environment. Nat Rev Microbiol. (2022) 20:257–69. doi: 10.1038/s41579-021-00649-x, 34737424 PMC8567979

[43] Holm G Snape JR Murray-Smith R Talbot J Taylor D Sörme P. Implementing Ecopharmacovigilance in practice: challenges and potential opportunities. Drug Saf. (2013) 36:533–46. doi: 10.1007/s40264-013-0049-3, 23620169 PMC3691479

[44] Xu W Pan Z Wu Y An XL Wang W Adamovich B . A database on the abundance of environmental antibiotic resistance genes. Sci Data. (2024) 11:250. doi: 10.1038/s41597-024-03084-8, 38413616 PMC10899624

[45] Zheng D Yin G Liu M Hou L Yang Y Van Boeckel TP . Global biogeography and projection of soil antibiotic resistance genes. Sci Adv. (2022) 8:eabq8015. doi: 10.1126/sciadv.abq8015, 36383677 PMC9668297

[46] Hendriksen RS Munk P Njage P van Bunnik B McNally L Lukjancenko O . Global monitoring of antimicrobial resistance based on metagenomics analyses of urban sewage. Nat Commun. (2019) 10:1124. doi: 10.1038/s41467-019-08853-3, 30850636 PMC6408512

[47] Willms IM Yuan J Penone C Goldmann K Vogt J Wubet T . Distribution of medically relevant antibiotic resistance genes and mobile genetic elements in soils of temperate forests and grasslands varying in land use. Genes (Basel). (2020) 11:150. doi: 10.3390/genes11020150, 32019196 PMC7073645

[48] Delgado-Baquerizo M Hu HW Maestre FT Guerra CA Eisenhauer N Eldridge DJ . The global distribution and environmental drivers of the soil antibiotic resistome. Microbiome. (2022) 10:219. doi: 10.1186/s40168-022-01405-w, 36503688 PMC9743735

[49] Brand JS Gauffin O Sartori D Fusaroli M Sköld H Bergvall T . VigiBase: resource profile update with a summary of global patterns and trends in adverse event reports for medicines and vaccines. Drug Saf. (2026). doi: 10.1007/s40264-025-01642-6 PMC13160990 41618069

[50] Mitchell J Lundquist P Westerberg C Purohit M Stålsby Lundborg CA. ‘One Health’ Cross-Sectional Analysis of Reports of Potential Antibiotic Resistance Cases in International Pharmacovigilance Databases. Front Public Health. (2026) 14. doi: 10.3389/fpubh.2026.1799220 40480533

[51] Mitchell J Maza Larrea J Dzidzornu ED Pandit J. Ecopharmacovigilance and pharmacovigilance: an analysis of environment-related reporting in VigiBase. Environ Sci Pollut Res. (2026) 33:6816–6826. 10.1007/s11356-026-37744-6 PMC13124797 41986825

