# Supplementary material for: A comparison of antibiotic resistance reports in pharmacovigilance databases and conventional surveillance across “One Health”
Source: Front Public Health. 2026 May 15;14:1758180. doi: 10.3389/fpubh.2026.1758180 (PMC13219042; doi:10.3389/fpubh.2026.1758180)
Supplement: Supplementary file 1 [file Data_Sheet_1.docx]

**Supplementary Material**

Supplementary Table 1. MedDRA and VeDDRA Preferred Terms used to identify cases of potential antibiotic resistance and environmental exposure in VigiBase and EudraVigilance Veterinary.

| Potential Antibiotic Resistance Terms MedDRA | |
| --- | --- |
| “Probable” | **“Possible”** |
| Drug tolerance*, Drug tolerance increased*, Multiple-drug resistance*, Drug resistance*, Pathogen resistance*, Antimicrobial susceptibility test resistant*, Antimicrobial susceptibility test intermediate* | Absence of immediate treatment response, Atypical dose response relationship, Drug effect less than expected, Drug ineffective, Drug ineffective for unapproved indication, Loss of therapeutic response, Missing dose response relationship, Paradoxical drug reaction, Remission not achieved, Therapeutic product effect decreased, Therapeutic product effect incomplete, Therapeutic product effect variable, Therapeutic product ineffective, Therapeutic product ineffective for unapproved indication, Therapeutic response changed, Therapeutic response decreased, Therapy non-responder, Therapy partial responder, Treatment failure |
| Potential Antibiotic Resistance Terms VeDDRA | |
| Lack of efficacy | |
| Environmental-related Preferred Terms MedDRA | |
| Environmental exposure, Exposure to chemical pollution, Exposure to contaminated air, Exposure to contaminated water, Exposure to polluted soil, Flooding, Food contamination, Pollution, Poor sanitation, Water pollution, Idiopathic environmental intolerance | |
| Environment-related Preferred Terms VeDDRA | |
| Environmental Incidence | |

**Traditional Antibiotic Resistance**

Supplementary Table 2. Total number of resistant tests from the Global Antimicrobial Resistance and Use Surveillance System (GLASS) data in 2022, for each source of infection, by continent and ATC third level

|  |  | Bloodstream | | | Gastrointestinal | | | Gonorrhoea | | | UTIs | | | Total | | |
| --- | --- | --- | --- | --- | --- | --- | --- | --- | --- | --- | --- | --- | --- | --- | --- | --- |
| **ATC Third Level** | **Continent** | **N = Tests** | **N = Positive** | **%** | **N = Tests** | **N = Positive** | **%** | **N = Tests** | **N = Positive** | **%** | **N = Tests** | **N = Positive** | **%** | **N = Tests** | **N = Positive** | **%** |
| J01A | Africa | 4,346 | 169 | 3.9 | 0 | 0 |  | 0 | 0 |  | 0 | 0 |  | 4,346 | 169 | 3.9 |
| J01A | Americas | 1,155 | 91 | 7.9 | 0 | 0 |  | 0 | 0 |  | 0 | 0 |  | 1,155 | 91 | 7.9 |
| J01A | Asia | 11,974 | 2,131 | 17.8 | 0 | 0 |  | 0 | 0 |  | 0 | 0 |  | 11,974 | 2,131 | 17.8 |
| J01A | Europe | 0 | 0 |  | 0 | 0 |  | 0 | 0 |  | 0 | 0 |  | 0 | 0 |  |
| J01A | Oceania | 0 | 0 |  | 0 | 0 |  | 0 | 0 |  | 0 | 0 |  | 0 | 0 |  |
| **ppl** |  | **17,475** | **2,391** | **13.7** | **0** | **0** |  | **0** | **0** |  | **0** | **0** |  | **17,475** | **2,391** | **13.7** |
| J01C | Africa | 775 | 442 | 57.0 | 0 | 0 |  | 0 | 0 |  | 1,274 | 1,167 | 91.6 | 2,049 | 1,609 | 78.5 |
| J01C | Americas | 2,346 | 305 | 13.0 | 0 | 0 |  | 0 | 0 |  | 498 | 237 | 47.6 | 2,844 | 542 | 19.1 |
| J01C | Asia | 93,994 | 37,163 | 39.5 | 0 | 0 |  | 0 | 0 |  | 416,218 | 178,561 | 42.9 | 510,212 | 21,5724 | 42.3 |
| J01C | Europe | 17,086 | 572 | 3.3 | 0 | 0 |  | 0 | 0 |  | 0 | 0 |  | 17,086 | 572 | 3.3 |
| J01C | Oceania | 521 | 6 | 1.2 | 0 | 0 |  | 0 | 0 |  | 0 | 0 |  | 521 | 6 | 1.2 |
| **J01C** |  | **114,722** | **38,488** | **33.5** | **0** | **0** |  | **0** | **0** |  | **417,990** | **179,965** | **43.1** | **532,712** | **21,8453** | **41.0** |
| J01D | Africa | 109,058 | 43,659 | 40.0 | 2280 | 119 | 5.2 | 921 | 18 | 2.0 | 343,245 | 48,178 | 14.0 | 455,504 | 91,974 | 20.2 |
| J01D | Americas | 179,856 | 28,719 | 16.0 | 9,805 | 465 | 4.7 | 11,668 | 19 | 0.2 | 1,498,908 | 131,907 | 8.8 | 1,700,237 | 161,110 | 9.5 |
| J01D | Asia | 1,135,157 | 231,809 | 20.4 | 12,620 | 864 | 6.8 | 2,808 | 275 | 9.8 | 4,261,237 | 681,439 | 16.0 | 5,411,822 | 914,387 | 16.9 |
| J01D | Europe | 1,121,216 | 117,715 | 10.5 | 33,244 | 738 | 2.2 | 7,701 | 20 | 0.3 | 4,860,485 | 209,428 | 4.3 | 6,022,646 | 327,901 | 5.4 |
| J01D | Oceania | 34,387 | 2,039 | 5.9 | 6,334 | 42 | 0.7 | 4,643 | 27 | 0.6 | 384,454 | 21,683 | 5.6 | 429,818 | 23,791 | 5.5 |
| **J01D** |  | **2,579,674** | **423,941** | **16.4** | **64,283** | **2,228** | **3.5** | **27,741** | **359** | **1.3** | **11,348,329** | **1,092,635** | **9.6** | **14,020,027** | **1,519,163** | **10.8** |
| J01E | Africa | 15,402 | 10,791 | 70.1 | 0 | 0 |  | 0 | 0 |  | 60,788 | 33,971 | 55.9 | 76,190 | 44,762 | 58.8 |
| J01E | Americas | 20,627 | 9,203 | 44.6 | 0 | 0 |  | 0 | 0 |  | 284,604 | 117,235 | 41.2 | 305,231 | 126,438 | 41.4 |
| J01E | Asia | 42,384 | 22,206 | 52.4 | 0 | 0 |  | 0 | 0 |  | 320,006 | 153,522 | 48.0 | 362,390 | 175,728 | 48.5 |
| J01E | Europe | 1,091 | 360 | 33.0 | 0 | 0 |  | 0 | 0 |  | 1,027,669 | 184,514 | 18.0 | 1,028,760 | 184,874 | 18.0 |
| J01E | Oceania | 6,929 | 1,694 | 24.4 | 0 | 0 |  | 0 | 0 |  | 82,433 | 17,817 | 21.6 | 89,362 | 19,511 | 21.8 |
| **J01E** |  | **86,433** | **44,254** | **51.2** | **0** | **0** |  | **0** | **0** |  | **1,775,500** | **507,059** | **28.6** | **1,861,933** | **551,313** | **29.6** |
| J01F | Africa | 0 | 0 |  | 25 | 3 | 12.0 | 486 | 42 | 8.6 | 0 | 0 |  | 511 | 45 | 8.8 |
| J01F | Americas | 0 | 0 |  | 1278 | 25 | 2.0 | 5,964 | 322 | 5.4 | 0 | 0 |  | 7,242 | 347 | 4.8 |
| J01F | Asia | 0 | 0 |  | 117 | 1 | 0.9 | 1,468 | 203 | 13.8 | 0 | 0 |  | 1,585 | 204 | 12.9 |
| J01F | Europe | 0 | 0 |  | 200 | 75 | 37.5 | 3,211 | 575 | 17.9 | 0 | 0 |  | 3,411 | 650 | 19.1 |
| J01F | Oceania | 0 | 0 |  | 16 | 4 | 25.0 | 4,643 | 147 | 3.2 | 0 | 0 |  | 4,659 | 151 | 3.2 |
| **J01F** |  | **0** | **0** |  | **1636** | **108** | **6.6** | **15,772** | **1,289** | **8.2** | **0** | **0** |  | **17,408** | **1,397** | **8.0** |
| J01G | Africa | 10,344 | 6,220 | 60.1 | 0 | 0 |  | 362 | 0 | 0.0 | 0 | 0 |  | 10,706 | 6,220 | 58.1 |
| J01G | Americas | 3,941 | 1,056 | 26.8 | 0 | 0 |  | 3,742 | 23 | 0.6 | 0 | 0 |  | 7,683 | 1,079 | 14.0 |
| J01G | Asia | 41,735 | 22,242 | 53.3 | 0 | 0 |  | 241 | 0 | 0.0 | 0 | 0 |  | 41,976 | 22,242 | 53.0 |
| J01G | Europe | 16,938 | 10,122 | 59.8 | 0 | 0 |  | 0 | 0 |  | 0 | 0 |  | 16,938 | 10,122 | 59.8 |
| J01G | Oceania | 197 | 4 | 2.0 | 0 | 0 |  | 2,278 | 0 | 0.0 | 0 | 0 |  | 2,475 | 4 | 0.2 |
| **J01G** |  | **73,155** | **39,644** | **54.2** | **0** | **0** |  | **6,623** | **23** | **0.3** | **0** | **0** |  | **79,778** | **39,667** | **49.7** |
| J01M | Africa | 17,804 | 8,220 | 46.2 | 1364 | 90 | 6.6 | 42 | 42 | 100.0 | 66,958 | 21,263 | 31.8 | 86,168 | 29,615 | 34.4 |
| J01M | Americas | 38,571 | 12,898 | 33.4 | 7514 | 1,431 | 19.0 | 5,967 | 2,420 | 40.6 | 378,246 | 123,708 | 32.7 | 430,298 | 140,457 | 32.6 |
| J01M | Asia | 285,191 | 92,791 | 32.5 | 6367 | 617 | 9.7 | 1,814 | 1,507 | 83.1 | 1,216,053 | 442,951 | 36.4 | 1,509,425 | 537,866 | 35.6 |
| J01M | Europe | 270,376 | 55,671 | 20.6 | 14289 | 2,559 | 17.9 | 3,211 | 1,785 | 55.6 | 2,236,457 | 244,200 | 10.9 | 2,524,333 | 304,215 | 12.1 |
| J01M | Oceania | 6,785 | 875 | 12.9 | 2134 | 77 | 3.6 | 4,643 | 2,515 | 54.2 | 82,871 | 9,089 | 11.0 | 96,433 | 12,556 | 13.0 |
| **J01M** |  | **618,727** | **170,455** | **27.5** | **31668** | **4,774** | **15.1** | **15,677** | **8,269** | **52.7** | **3,980,585** | **841,211** | **21.1** | **4,646,657** | **1,024,709** | **22.1** |
| J01X | Africa | 1,094 | 44 | 4.0 | 0 | 0 |  | 128 | 7 | 5.5 | 3,921 | 97 | 2.5 | 5,143 | 148 | 2.9 |
| J01X | Americas | 8,212 | 280 | 3.4 | 0 | 0 |  | 1,740 | 0 | 0.0 | 43,767 | 1,192 | 2.7 | 53,719 | 1,472 | 2.7 |
| J01X | Asia | 33,855 | 1,634 | 4.8 | 0 | 0 |  | 1,255 | 0 | 0.0 | 61,088 | 1,266 | 2.1 | 96,198 | 2,900 | 3.0 |
| J01X | Europe | 28,770 | 1,808 | 6.3 | 0 | 0 |  | 2,513 | 0 | 0.0 | 992 | 7 | 0.7 | 32,275 | 1,815 | 5.6 |
| J01X | Oceania | 764 | 0 | 0.0 | 0 | 0 |  | 4,251 | 0 | 0.0 | 0 | 0 | 0.0 | 5,015 | 0 | 0.0 |
| **J01X** |  | **72,695** | **3,766** | **5.2** | **0** | **0** |  | **9,887** | **7** | **0.1** | **109,768** | **2,562** | **2.3** | **192,350** | **6,335** | **3.3** |

Abbreviations: ATC – Anatomical Therapeutic Chemical; J01A – tetracyclines; J01B – amphenicols; J01C – beta-lactam antibacterials, penicillins; J01D – other beta-lactam antibacterials; J01E sulfonamides and trimethoprim; J01F – macrolides, lincosamides and streptogramins; J01G – aminoglycoside antibacterials; J01M – quinolone antibacterials; J01R – combination of antibacterials, J01W – herbal antibacterials; J01X – other antibacterials.

Supplementary Table 3. Total number of tests resistant tests from the EFSA/ECDC joint report on antibiotic resistance 2021/2022 for each source of infection, by continent and ATC third level

|  |  | Escherichia coli | | | Campylobacter spp. | | |
| --- | --- | --- | --- | --- | --- | --- | --- |
| ABX | ATC | Tests | Resistant Tests | % Resistance | Tests | Resistant Tests | % Resistance |
| TGC | J01AA | 7051 | 65 | 0.9 | 0 |  |  |
| TET | J01AA | 7051 | 2790 | 39.6 | 7747 | 4988 | 64.4 |
| CHL | J01BA | 7051 | 938 | 13.3 | 7747 | 20 | 0.3 |
| AMP | J01CA | 7051 | 3315 | 47.0 | 0 |  |  |
| CTX | J01DD | 7051 | 93 | 1.3 | 0 |  |  |
| CAZ | J01DD | 7051 | 85 | 1.2 | 0 |  |  |
| MEM | J01DH | 7051 | 1 | 0.0 | 0 |  |  |
| ETP | J01DH | 0 |  |  | 7747 | 2060 | 26.6 |
| TMP | J01EA | 7051 | 1909 | 27.1 | 0 |  |  |
| SMX | J01EQ | 7051 | 2452 | 34.8 | 0 |  |  |
| AZM | J01FA | 7051 | 140 | 2.0 | 0 |  |  |
| ERY | J01FA | 0 |  |  | 7747 | 606 | 7.8 |
| GEN | J01GB | 7051 | 348 | 4.9 | 7747 | 72 | 0.9 |
| AMK | J01GB | 7051 | 7 | 0.1 | 0 |  |  |
| CIP | J01MA | 7051 | 3020 | 42.8 | 7747 | 5567 | 71.9 |
| NAL | J01MB | 7051 | 2671 | 37.9 | 0 |  |  |
| COL | J01XB | 7051 | 106 | 1.5 | 0 |  |  |

Abbreviations: EFSA – European Food Safety Authority, ECDC - European Centre for Disease Prevention and Control, ABX – antibiotic, ATC – Anatomical Therapeutic Chemical GEN - Gentamicin, AMK - Amikacin, CHL – Chloramphenicol, AMP – Ampicillin, CTX – cefotaxime, CAZ – Ceftazidime, MEM – Meropenem, TGC - Tigecycline, NAL - Nalidixic acid, CIP – Ciprofloxacin, AZM – Azithromycin, COL – Colistin, SMX – Sulfamethoxazole, TMP – Trimethoprim, TET - Tetracycline, ETP – Ertapenem, ERY- erythromycin

**Pharmacovigilance**

Supplementary Table 3. Total number of reported antibiotics for each ATC third level group for reports in VigiBase of potential antibiotic resistance cases by continent.

| **Continent** | **ATC** | **Count** | **Total Count for the Continent** | **Percentage of Reports in the Continent** |
| --- | --- | --- | --- | --- |
| Africa | J01A | 12 | 1602 | 0.7 |
| Americas | J01A | 1979 | 22296 | 8.9 |
| Asia | J01A | 159 | 4010 | 4.0 |
| Europe | J01A | 493 | 9868 | 5.0 |
| Oceania | J01A | 55 | 700 | 7.9 |
| Africa | J01B | 2 | 1602 | 0.1 |
| Americas | J01B | 40 | 22296 | 0.2 |
| Asia | J01B | 13 | 4010 | 0.3 |
| Europe | J01B | 25 | 9868 | 0.3 |
| Oceania | J01B | 8 | 700 | 1.1 |
| Africa | J01C | 565 | 1602 | 35.3 |
| Americas | J01C | 2250 | 22296 | 10.1 |
| Asia | J01C | 735 | 4010 | 18.3 |
| Europe | J01C | 1834 | 9868 | 18.6 |
| Oceania | J01C | 105 | 700 | 15.0 |
| Africa | J01D | 265 | 1602 | 16.5 |
| Americas | J01D | 3280 | 22296 | 14.7 |
| Asia | J01D | 1079 | 4010 | 26.9 |
| Europe | J01D | 1816 | 9868 | 18.4 |
| Oceania | J01D | 127 | 700 | 18.1 |
| Africa | J01E | 14 | 1602 | 0.9 |
| Americas | J01E | 976 | 22296 | 4.4 |
| Asia | J01E | 124 | 4010 | 3.1 |
| Europe | J01E | 316 | 9868 | 3.2 |
| Oceania | J01E | 36 | 700 | 5.1 |
| Africa | J01F | 57 | 1602 | 3.6 |
| Americas | J01F | 3843 | 22296 | 17.2 |
| Asia | J01F | 493 | 4010 | 12.3 |
| Europe | J01F | 1156 | 9868 | 11.7 |
| Oceania | J01F | 131 | 700 | 18.7 |
| Africa | J01G | 340 | 1602 | 21.2 |
| Americas | J01G | 1042 | 22296 | 4.7 |
| Asia | J01G | 238 | 4010 | 5.9 |
| Europe | J01G | 509 | 9868 | 5.2 |
| Oceania | J01G | 31 | 700 | 4.4 |
| Africa | J01M | 81 | 1602 | 5.1 |
| Americas | J01M | 3121 | 22296 | 14.0 |
| Asia | J01M | 467 | 4010 | 11.6 |
| Europe | J01M | 1235 | 9868 | 12.5 |
| Oceania | J01M | 63 | 700 | 9.0 |
| Africa | J01R | 1 | 1602 | 0.1 |
| Americas | J01R | 51 | 22296 | 0.2 |
| Asia | J01R | 17 | 4010 | 0.4 |
| Europe | J01R | 95 | 9868 | 1.0 |
| Americas | J01W | 31 | 22296 | 0.1 |
| Asia | J01W | 16 | 4010 | 0.4 |
| Europe | J01W | 17 | 9868 | 0.2 |
| Africa | J01X | 265 | 1602 | 16.5 |
| Americas | J01X | 5683 | 22296 | 25.5 |
| Asia | J01X | 669 | 4010 | 16.7 |
| Europe | J01X | 2372 | 9868 | 24.0 |
| Oceania | J01X | 144 | 700 | 20.6 |

Abbreviations: ATC – Anatomical Therapeutic Chemical; J01A – tetracyclines; J01B – amphenicols; J01C – beta-lactam antibacterials, penicillins; J01D – other beta-lactam antibacterials; J01E sulfonamides and trimethoprim; J01F – macrolides, lincosamides and streptogramins; J01G – aminoglycoside antibacterials; J01M – quinolone antibacterials; J01R – combination of antibacterials, J01W – herbal antibacterials; J01X – other antibacterials.

Supplementary Table 4. Total number of reported antibiotics for each ATC third level group for reports in EudraVigilance Veterinary of potential antibiotic resistance cases, by groupings of animal species.

| ATCvet | All | | Food producing | | Matching species | |
| --- | --- | --- | --- | --- | --- | --- |
|  | Frequency | Percentage | Frequency | Percentage | Frequency | Percentage |
| J01A | 135 | 10.158014 | 112 | 10.810811 | 90 | 9.473684 |
| J01B | 111 | 8.352144 | 107 | 10.328185 | 98 | 10.315789 |
| J01C | 334 | 25.131678 | 235 | 22.683398 | 216 | 22.736842 |
| J01D | 126 | 9.480813 | 72 | 6.949807 | 71 | 7.473684 |
| J01E | 74 | 5.568096 | 64 | 6.17706 | 63 | 6.631579 |
| J01F | 270 | 20.316027 | 256 | 24.710425 | 240 | 25.263158 |
| J01G | 56 | 4.213695 | 47 | 4.536680 | 44 | 4.631579 |
| J01M | 80 | 6.019564 | 46 | 4.440154 | 43 | 4.526316 |
| J01R | 48 | 3.611738 | 44 | 4.247104 | 44 | 4.631579 |
| J01X | 95 | 7.148232 | 53 | 5.115830 | 41 | 4.315789 |

Abbreviations: ATC – Anatomical Therapeutic Chemical; J01A – tetracyclines; J01B – amphenicols; J01C – beta-lactam antibacterials, penicillins; J01D – other beta-lactam antibacterials; J01E sulfonamides and trimethoprim; J01F – macrolides, lincosamides and streptogramins; J01G – aminoglycoside antibacterials; J01M – quinolone antibacterials; J01R – combination of antibacterials, J01W – herbal antibacterials; J01X – other antibacterials.
