# Supplementary material for: A comparison of antibiotic resistance reports in pharmacovigilance databases and conventional surveillance across “One Health”
Source: Front Public Health. 2026 May 15;14:1758180. doi: 10.3389/fpubh.2026.1758180 (PMC13219042; doi:10.3389/fpubh.2026.1758180)
Supplement: Supplementary file 2 [file Image_1.pdf]

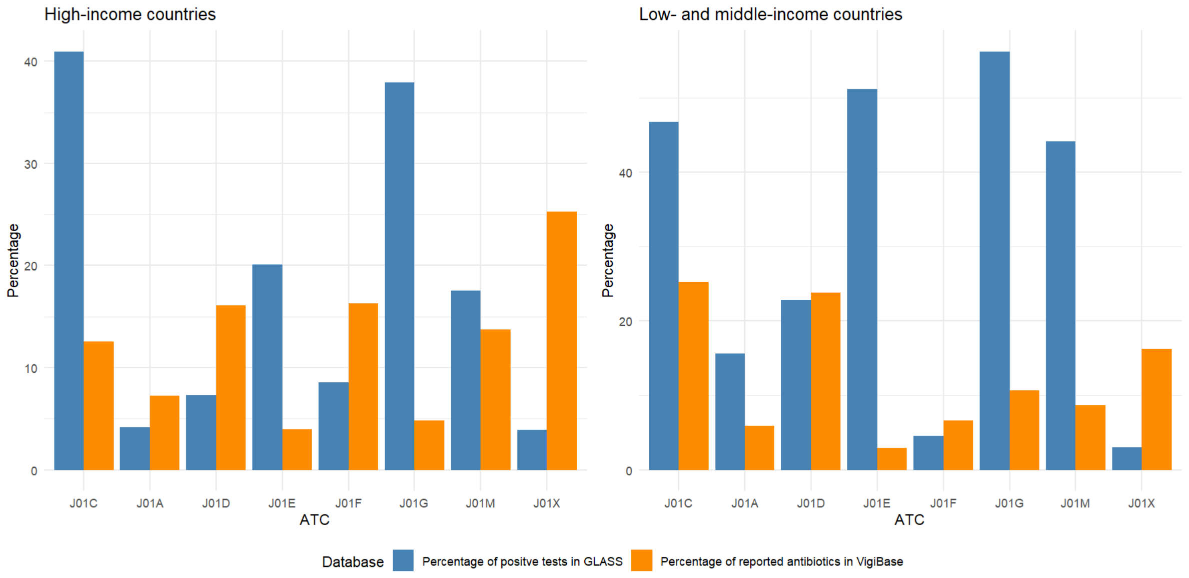

Supplementary Figure 1. Percentage of resistant tests in GLASS for all sources of infection and the percentage of reported antibiotics in potential cases of antibiotic resistance in VigiBase for each ATC third group level, for all reports from high-income countries and low- and middle-income countries separately. Number of antibiotics included from VigiBase reports for each graph – High-income n=32,621; Low- and middle-income n=5,539. Number of tests reported to GLASS for each graph – High-income n=16,605,129; Low- and middle-income n=4,763,211.

Abbreviations: ATC – Anatomical Therapeutic Chemical; J01A – tetracyclines; J01B – amphenicols; J01C – beta-lactam antibacterials, penicillins; J01D – other beta-lactam antibacterials; J01E sulfonamides and trimethoprim; J01F – macrolides, lincosamides and streptogramins; J01G – aminoglycoside antibacterials; J01M – quinolone antibacterials; J01R – combination of antibacterials, J01W – herbal antibacterials; J01X – other antibacterials.
