# Supplementary material for: A comparison of antibiotic resistance reports in pharmacovigilance databases and conventional surveillance across “One Health”
Source: Front Public Health. 2026 May 15;14:1758180. doi: 10.3389/fpubh.2026.1758180 (PMC13219042; doi:10.3389/fpubh.2026.1758180)
Supplement: Supplementary file 3 [file Image_2.pdf]

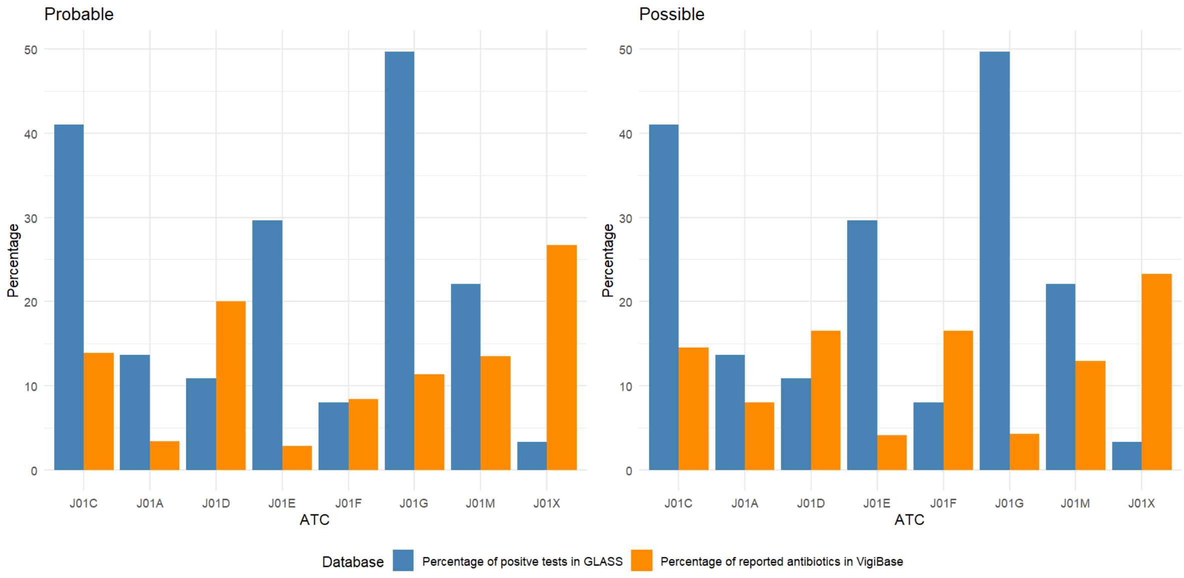

Supplementary Figure 2. Percentage of resistant tests in GLASS for all sources of infection and the percentage of reported antibiotics in potential cases of antibiotic resistance in VigiBase for each ATC third group level, for all reports grouped by likelihood of the VigiBase reports being cases of antibiotic resistance cases. Number of antibiotics included from VigiBase reports for each graph – Probable n=7,668; Possible n=30,492. Number of tests reported to GLASS n=21,368,340.

Abbreviations: ATC – Anatomical Therapeutic Chemical; J01A – tetracyclines; J01B – amphenicols; J01C – beta-lactam antibacterials, penicillins; J01D – other beta-lactam antibacterials; J01E sulfonamides and trimethoprim; J01F – macrolides, lincosamides and streptogramins; J01G – aminoglycoside antibacterials; J01M – quinolone antibacterials; J01R – combination of antibacterials, J01W – herbal antibacterials; J01X – other antibacterials.
